# β-catenin inhibitors in cancer therapeutics: intricacies and way forward

**DOI:** 10.1080/21655979.2023.2251696

**Published:** 2023-09-01

**Authors:** Arundhathi Dev, Meenakshi Vachher, Chandra Prakash Prasad

**Affiliations:** a Department of Medical Oncology (Laboratory), DR BRAIRCH, All India Institute of Medical Sciences, New Delhi, India; b Department of Biochemistry, Institute of Home Economics, University of Delhi, New Delhi, India

**Keywords:** β-catenin, cancer, WNT signaling, mutations, β-catenin inhibitors, natural compounds

## Abstract

β-catenin is an evolutionary conserved, quintessential, multifaceted protein that plays vital roles in cellular homeostasis, embryonic development, organogenesis, stem cell maintenance, cell proliferation, migration, differentiation, apoptosis, and pathogenesis of various human diseases including cancer. β-catenin manifests both signaling and adhesive features. It acts as a pivotal player in intracellular signaling as a component of versatile WNT signaling cascade involved in embryonic development, homeostasis as well as in carcinogenesis. It is also involved in Ca^2+^ dependent cell adhesion via interaction with E-cadherin at the adherens junctions. Aberrant β-catenin expression and its nuclear accumulation promote the transcription of various oncogenes including c-Myc and cyclinD1, thereby contributing to tumor initiation, development, and progression. β-catenin’s expression is closely regulated at various levels including its stability, sub-cellular localization, as well as transcriptional activity. Understanding the molecular mechanisms of regulation of β-catenin and its atypical expression will provide researchers not only the novel insights into the pathogenesis and progression of cancer but also will help in deciphering new therapeutic avenues. In the present review, we have summarized the dual functions of β-catenin, its role in signaling, associated mutations as well as its role in carcinogenesis and tumor progression of various cancers. Additionally, we have discussed the challenges associated with targeting β-catenin molecule with the presently available drugs and suggested the possible way forward in designing new therapeutic alternatives against this oncogene.

## 1. Introduction

β-catenin is an 88 kDa versatile, multifunctional, and evolutionary-conserved protein known to contribute to the regulation of cell fate and development processes all through the lifespan of animals. It is identified as the *Drosophila* homolog of *armadillo (ARM)* and belongs to the *ARM* family of proteins. It is a seminal component of the WNT signaling pathway, which is well studied, and demonstrated to be involved in the establishment of body axis and orchestration of tissue and organ development during embryonic growth [1]. Even in adult organs, it contributes significantly to processes like tissue homeostasis, organogenesis, stem cell maintenance, migration, apoptosis, proliferation, cell renewal, repair, and regeneration [2,3]. Given its functional significance, the aberrant expression of β-catenin has been widely regarded to play crucial roles in malignant transformation, progression, and metastasis. Its implications in carcinogenesis notwithstanding, advances in therapeutic interventions for targeting β-catenin molecule are still nascent. The present review attempts to comprehensively discuss the molecular mechanisms as well as evaluate the existing therapeutic strategies aimed at addressing the clinical burden posed by deregulated β-catenin in cancers.

## 2. Discovery and dual functionality

β-catenin is a well-recognized key effector of the WNT signaling pathway, which was initially discovered for its structural role in cellular adhesion. In 1989, Ozawa et al. initially investigated the structural association of β–catenin along with α-catenin and γ-catenin with the cytoplasmic domain of Ca^2+^dependent cell adhesion molecule uvomorulin, which was later renamed E-cadherin [4]. The three proteins were named α, β, and γ-catenins (from ‘*Catena*’ meaning chain in Latin) indicating their potential role in anchoring E-cadherin to the cytoskeleton structures.

The potential role of β-catenin in cellular signaling was deciphered using a series of developmental studies in *Drosophila* wherein the *armadillo (ARM)* gene, was identified as a key segment polarity gene in early patterning and morphogenetic screens investigating mutations influencing embryonic development [5]. *ARM* mutants were found to bear resemblance to Wingless *(Wg)* null mutant’s embryonic phenotype. *Wg* is a secreted extracellular protein that affects the determination of cell fate beyond its site of production along its morphogen gradient. However, the transformations seen in *ARM* embryos were authenticated to be cell autonomous, suggesting that *ARM* acted downstream to wingless. Epistatic analysis revealed that the *armadillo’s* segment polarity function was regulated by wingless [6], which proved to be a turning point leading to a series of investigational studies identifying other components (like Disheveled, APC, Axin, etc.) and interactors leading to the characterization of the WNT-β-catenin or the Wingless-Armadillo signaling pathway [7].

Analysis of β-catenin conditional gain and loss of function mutant mice further revealed that canonical WNT signals control progenitor cell expansion decide lineage in the early embryo and various adult organs, thereby emphasizing its crucial role in the development as well as a disease [8]. β-catenin involvement in the fundamental processes of adhesion and signaling makes it a poster child for the phenomenon of ‘Moonlighting,’ especially in the context of cancer [9]. While cellular adhesion and signaling might seem to be unrelated, the fact that these processes share an evolutionarily conserved key protein hints at a requirement for a synchronized control between them. The relevance of such a coordinated control is apparent in the phenomenon of Epithelial–Mesenchymal Transition (EMT), a key event in cancer metastasis that is characterized by a rewiring of the cellular signaling accompanied by loss of adhesion.

The obvious question that arises is how the β-catenin molecule efficiently pivots between two distinct processes. The answer to these intricacies of multi-functionality exhibited by β-catenin lies in its structure, specific phosphorylation mechanisms, competition among interacting partners, and spatio-temporal segregation.

## 3. β-catenin structural composition

**Figure 1.**
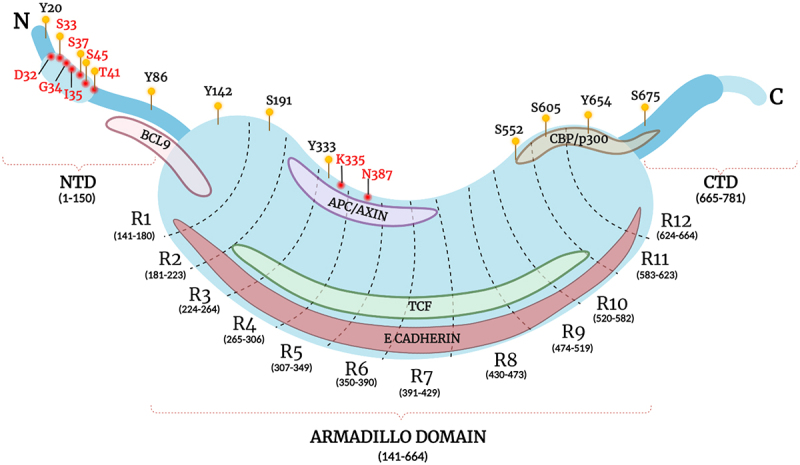
Schematic representation of β-catenin protein structure.

Notes: The diagram encapsulates the principal structural breakdown of β-catenin where the three domains – N terminal domain (amino acids 1-150), *armadillo* domain with *armadillo* repeat R1 to R12 (amino acids 141-664), and C terminal domain (amino acids 665-781) along with the major binding regions for E-cadherin, TCF, BCL9, APC/AXIN, and CBP/P300 in the respective domains are marked. The main phosphorylated residues (denoted using symbol “”) and most frequently mutated residues in various cancers (denoted using symbol “”) are also included in the corresponding domains. Created with BioRender.com

β-catenin is composed of 781 amino acid (aa) residues having three major domains with distinctive charge distributions. The amino-terminal domain (NTD) constitutes approximately the first 130 aa residues followed by the central core region of about 550 aa forming the *ARM* repeat domain, the most conserved region of the β-catenin protein. This region has 12 imperfect *ARM* sequence repeats (R1–12), where each *ARM* repeat constitutes a tandem repeat of about 42 aa forming a motif of three α-helices forming a triangular shape [10]. The 12 repeats together to form a compact super helix featuring a long positively charged groove spanning the entire domain and contains the binding site for almost 20 or so β-catenin interacting partners [11]. Various β-catenin binding partners share overlapping binding sites in the groove of the central region, explaining the mutual exclusivity of the key interactors including E-cadherin, adenomatous polyposis coli (APC), and T-cell factor/lymphoid enhancer factor (TCF/LEF). The central core is flanked at the terminal end by the carboxyl terminal domain (CTD) of approximately 100 aa [12]. The sequence of the terminal domains is less conserved than the *ARM* repeat domain. Additionally, they are also structurally flexible, and are known as intrinsically disordered protein regions (IDPRs). Though the exact mechanisms remain largely elusive, these disordered tails have unique roles in synergistically regulating the interaction patterns of β-catenin [13,14]. It is also noteworthy that the majority of cancer-associated mutations on β-catenin are found in the NTD, underlining the regulatory role played by this domain [15]. Schematic illustration of β-catenin protein structure is provided in Figure 1.

## 4. The tightrope between adhesion and signaling

The commitment to signal transduction is modulated by the cytoplasmic pool of free β- catenin that includes newly synthesized protein or the unbound protein released from the adherens junctions [16]. The cytoplasmic levels of β-catenin are tightly controlled by phosphorylation-mediated degradation, which is chiefly orchestrated by the ‘Destruction complex’ (DC). This DC-multiprotein tertiary complex is formed by adenomatous polyposis coli (APC), Glycogen Synthase Kinase 3β (GSK3β), Casein Kinase 1α (CK1α), and APC axis inhibitor (AXIN), wherein AXIN and APC form the scaffold of the destruction complex [17]. A series of phosphorylation events are initiated by CK1α which phosphorylates β-catenin at the N-terminus at Ser45 followed by phosphorylation at Ser33, Ser37, and Thr41 by GSK3β [18]. Subsequently, phosphorylated β-catenin is recognized by β-transducin repeat containing protein (β-TrCP), an E3 ubiquitin ligase that catalyzes polyubiquitination upon forming a complex with Skp1 and Cullin, directing the protein toward the ubiquitin proteasome system (UPS) for degradation [19]. Previous studies have also identified several other E3 ubiquitin ligases (other than β-TrCP) for β-Catenin i.e. EDD [20], GID complex [21], Jade1 [22], Mule [23], SHPRH [24], and SIAH1 [25]. In an interesting study, Kitazawa et al. determined the rate-limiting factor for the formation of β-catenin DC by utilizing absolute protein quantification using LC-MS/MS [26]. Interaction of similar amounts of destruction complex-constituting proteins, and β-catenin resulted in 1468 destruction complex (DC) molecules per cell. Under steady-state conditions, the authors showed that the measured number of DCs was suitable for the control of the β-catenin destruction rate. Additionally, among all the DC-proteins, the amount of APC expression came out to be a rate-limiting factor for the formation of β-catenin DCs. Lybrand et al., using bimolecular fluorescence complementation (BiFC) in *Drosophila* showed that the cell nucleus is surrounded by active DCs which predominantly direct the β-catenin degradation and thereby its nuclear access [27]. However, such localization of DCs and its impact on β-catenin turnover in cancer cells need to be further investigated.

Thus, multiple mechanisms exist which inhibit β-catenin’s degradation, stabilizing it and promoting its nuclear translocation thereby eventually regulating its transcriptional activity. The major and most studied pathway involving β-catenin is the WNT signaling pathway.

## 5. Canonical WNT signaling

The WNT signaling is an evolutionarily conserved signaling which comprises canonical and non-canonical pathways that act either in a paracrine or autocrine manner. WNTs are a family of 19 secreted cysteine-rich glycoproteins of about 40 kDa that trigger intracellular signal transduction pathways upon binding to one of the 10 Frizzled (Fzd) receptors and an associated co-receptor [28]. β-catenin is considered as the ‘gatekeeper’ of the canonical WNT signaling [29]. A low WNT activity is often associated with enhanced functioning of the β-catenin DC. The WNT signaling is switched ‘ON’ when the extracellular WNT ligand binds to its G-protein coupled seven-span transmembrane receptor i.e. Fzd and co-receptors LRP5/6 causing them to multimerize at the cell surface [17]. The binding initiates not only a change in the conformation of the Fzd-LRP complex but also the CK1 or GSK3 mediated phosphorylation of the cytoplasmic tail of LRP, which then serves as a docking site for AXIN. The activated receptor multimer also recruits the protein disheveled (Dvl) that binds to the cytoplasmic end of Fzd [30]. The bound Dvl then starts a cascade of protein interactions interacting with and mobilizing AXIN-GSK3β-CK1-APC to the membrane and effectively sequestering and disrupting the complex. Despite several intensive studies, the exact mechanism of downregulation of the destruction of complex stability is still contended upon. The two competing strategies proposed- ‘inhibition of phosphorylation’ and ‘inhibition of ubiquitination’ are still extensively researched [31].

Notwithstanding, the mechanism of WNT stimulation results in a cytoplasmic accumulation of β-catenin which seems to be a prerequisite for its nuclear translocation. β-catenin does not contain the classical nuclear localization signal (NLS), and its import into the nucleus has been demonstrated to be independent of transport machinery-like importins or RAN-GTPase [29,32]. However, nuclear import/export chaperones like Smad3/4, LEF1, FOXM1, PAK4 [29,33], etc., have been shown to mediate the nuclear localization/retention of β-catenin in addition to its direct interaction with nuclear pore complex (NPC) [34].

Once inside the nucleus, β-catenin exerts transcriptional control over a larger number of gene targets despite lacking a DNA-binding domain. It functions as a transcriptional co-activator and binds to multiple transcription factors of which the best characterized is its interaction with the members of the TCF/LEF family [35]. The TCF/LEF complex has been experimentally shown to bind to the promoter-enhancer regions of their target genes. In the WNT ‘OFF’ state or when there is the absence of nuclear-localized β-catenin, the activity of the TCF/LEF complex is inhibited by the members of the Groucho/TLE, which are a family of co-repressors that maintain chromatin at a transcriptionally inactive state by associating with histone deacetylases. As the first step, nuclear β-catenin directly disrupts the repressor complex by displacing Groucho [36]. Following this, recruitment of the β-catenin ‘enhanceosome’ which includes BCL9, CBP, Pygopus, BRG1, etc., is observed [37]. Once activated, the transcriptional output of this complex includes c-Myc, CCND1, CDKN1A, cyclin D1, and Bcl-w, which are known to play critical roles in the process of carcinogenesis [38]. β-catenin can also interact with numerous other transcription factors like FOXO, HIF1, Sox family members, nuclear receptors, etc. leading to its diverse functions and cellular outputs [39]. The non-canonical pathways of WNT signaling are the WNT/Ca^2+^ pathway and the Planar Cell Polarity pathway [40]. Though they do not directly encompass the activation of β-catenin, studies have demonstrated crosstalk and coordinated integration between canonical and non-canonical pathways, where non-canonical WNTs can both activate β-catenin transcriptional activity and signal initiation of its GSK3β independent degradation depending on the cellular milieu [41,42]. Characteristic details of β-catenin enhanceosome is provided in **Box 1**.

## 6. β-catenin beyond WNTs

While canonical WNT signaling is pivotal to the regulation of β-catenin and thereby significant to development, cell homeostasis, and carcinogenesis; the regulation of transcriptional activity of β-catenin is not restricted to the WNT-mediated signalosome activation alone. The linear paradigm of signaling is sometimes inadequate in explaining the change in signaling dynamics over time and more importantly crosstalk with other pathways and networks. The other mechanisms that regulate β-catenin are briefly discussed below.

### 6.1. Receptor tyrosine kinases mediated post-translational modification (PTM)

Enhanced β-catenin signaling in response to EGFR-mediated tyrosine kinase phosphorylation was first reported in 1994, where EGF stimulation of epidermoid carcinoma cells showed a cytoplasmic accumulation and nuclear translocation of β-catenin followed by enhanced transcription of target genes such as *c-Myc*, along with its dissociation from the adherens junction [43]. Since then, various studies reported that EGF stimulation resulted in phosphorylation of β-catenin at Tyr20, Tyr86, Tyr142, and Tyr654 in a cell line and/or in context-dependent manner [44–46]. Stimulation with EGF was also shown to indirectly influence β-catenin post-translational modification and localization via activation of Akt which reportedly phosphorylated the protein at Ser552 [47]. In addition, the non-receptor Tyrosine kinase i.e. c-Src also phosphorylates β-catenin at Tyr333 augmenting its nuclear translocation and interaction with TCF7L2 and Pyruvate Kinase M2 in response to EGF stimulation [48].

Following EGF stimulation in HCT116 colon cancer cells, β-catenin was shown to interact with HDAC6 deacetylase leading to deacetylation at Lys49, inhibiting β-catenin’s phosphorylation at Ser45 and thus leading to nuclear localization [49]. Ultraviolet-induced EGFR activation has been shown to result in Tyr654 phosphorylation in β-catenin leading to dissociation of β-catenin from the adherens junctions, increased nuclear translocation, and transcriptional activation in keratinocytes [50]. IGF-1 or IGF-II can lead to the activation of Insulin-like growth factor 1 receptor (IGF1R) which disrupts cell–cell contacts leading to a simultaneous redistribution of E-cadherin and β-catenin from the adherens junctions to the cytosol and nucleus, promoting epithelial–mesenchymal transition (EMT) as well as cell migration [51]. In a study by Playford et al., IGF-I treatment augmented β-catenin Tyr99 phosphorylation and decreased its association with E-cadherin in colorectal cancer cell lines. β-catenin’s subcellular localization was altered from peripheral membrane to cytosolic with concomitant increased stability accompanied by increased GSK3β Ser9 phosphorylation [52]. Further, fibroblast growth factor-2 (FGF-2) has been shown to activate MAP kinase signaling which in turn phosphorylates β-catenin at Ser675 through MEKK2 that stabilizes β-catenin by recruiting deubiquitinase USP15 in osteoblasts [53].

Apart from tyrosine kinases, the interacting proteins of β-catenin also influence the signaling capacities of β-catenin. The following section deals with the modulation in the levels of β-catenin by virtue of its competitive interaction with the tumor suppressor gene i.e. PTEN and also the structural protein i.e. γ-catenin.

### 6.2. Interactors of β-catenin and their influence on signaling

Caveolae are lipid rafts enriched in caveolin-1 (CAV1) exhibiting invaginations of the membrane that mediate clathrin-independent endocytosis. CAV1 regulates this form of endocytosis and has been reported to act on AXIN as well as LRP6 that promotes the cytoplasmic enrichment of β-catenin, in a cell and context-dependent fashion [54]. Conde-Perez et al. hypothesized that since the CAV1 scaffolding domain interacts with both β-catenin and the tumor suppressor gene PTEN, there can be a molecular competition between the two proteins for CAV1 that might patronize its signaling outcomes [55].

**Figure 2.**
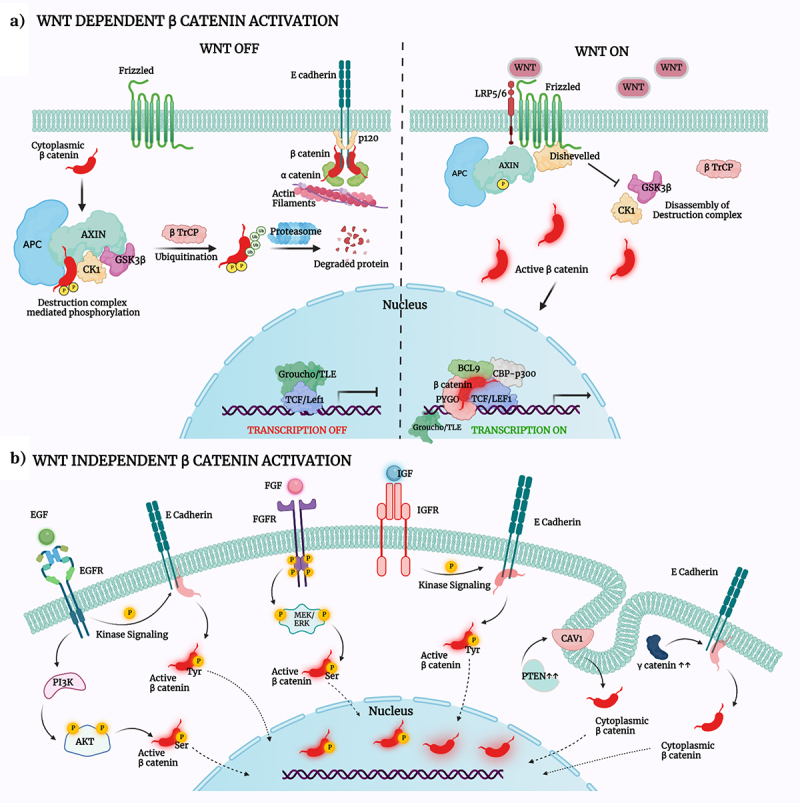
Mechanisms controlling β-catenin activation and dynamic localization.

Notes: Figure 2(a) schematically illustrates the regulation of β-catenin stability and localization by the canonical Wnt signaling pathway. In the Wnt ‘OFF’ state, the WNT ligands are absent and consequently, the entry of β-catenin into the nucleus is limited by the proteasomal degradation of β-catenin in the cytoplasm, which is facilitated by the phosphorylation of the protein by the AXIN-APC1-GSK3β-CK1 Destruction Complex (DC). Upon Activation, WNT ‘ON,’ the binding of WNT ligands to their frizzled receptor complex results in the recruitment of Disheveled and activates a series of events leading to disassembly of the destruction complex. This leads to cytoplasmic accumulation and subsequent translocation of β-catenin into the nucleus where it serves its transcriptional purpose. Figure 2(b) represents the WNT independent modes of activation of β-catenin that promotes its nuclear localization. It includes activating phosphorylation moderated by receptor tyrosine kinases like EGFR, FGFR, and IGFR. Competition of PTEN with β-catenin for CAV1 and competition of Plakoglobin (γ-catenin) for E-cadherin are also mechanisms that lead to the upregulation of β catenin in the cytoplasm, and subsequent nuclear translocation. Created with BioRender.com

Another competitor to β-catenin is γ-catenin/plakoglobin, a protein that shares structural homology and some functional similarity with β-catenin. In addition to the cadherins, γ-catenin has been demonstrated to bind to multiple other interactors of β-catenin including APC, GSK3β, TCF, LEF, AXIN, etc. [56]. In this background, Salomon et al. in 1997 showed an increased nuclear accumulation of β-catenin upon γ-catenin overexpression using fibrosarcoma cells [57]. This finding was subsequently reproduced in other cell lines and non-transformed cells where increased γ-catenin displaced β-catenin at the adherens junctions, consequently promoting its oncogenic signaling activity. Mechanisms regulating β-catenin activation and its localization are shown in Figure 2.

In essence, an elaborate understanding of β-catenin activation shows us that the transcriptional activity of the molecule is not restricted to only being a functional readout of the WNT pathway. Knowledge of the functional affiliations and crosstalk of protein is central in comprehending how the molecule is fine-tuned by cancer cells to promote proliferation, EMT, stemness, etc. In the following section, we take a glance at deregulation and its implications in malignancy.

## 7. β-catenin in cancers

Accumulating evidence suggests an unquestionable association between aberrant β-catenin expression and cancer development, hence establishing it as a cardinal molecule in carcinogenesis. β-catenin is known to be involved in cancer progression, invasion, as well as metastasis through the regulation of its transcriptional targets, including multiple oncogenes and tumor suppressor genes. The importance of β-catenin (independent of APC) in colon carcinogenesis gained traction after the discovery of mutations in the regulatory sequences of the β-catenin gene in APC wild-type colon cancers [58]. Mutant β-catenin is immune to APC modulated degradation, leading to its deregulated transcriptional activity and oncogenic function. Oncogenic mutations in β-catenin are infrequent but have been reported in multiple cancers like colorectal, melanoma, liver, and endometrial cancers [59]. Various mutations lead to different levels of WNT/β-catenin signaling influencing host cell homeostasis and might vary with different organs. Exon 3 (aa residue 5–80) of *CTNNB1* appears to be a mutation hotspot owing to a higher frequency of missense mutations in exon 3 which encodes for the regulatory site at the amino-terminal domain of β-catenin [15]. Interestingly, missense mutations (but not deletions) associated with *TP53* gene have been associated with cancer invasion by activating WNT/β-catenin signaling in mouse models of colorectal cancer [60,61]. Missense mutations in *TP53* are relatively common in human cancers giving rise to mutant p53 proteins with non-operational tumor suppressive activities [62,63]. Cancer cells harboring mutated p53 oncoproteins profoundly disrupt the nature of p53 pathway leading to tumor aggressiveness, invasion, and drug resistance. It has been observed that mutational inactivation of p53 is often associated with accumulation/activation of β-catenin in various cancers [64]. Sandot et al. reported that overexpression of wild-type p53 resulted in decreased expression of β-catenin in human and mouse cells [64]. p53 expression resulted in higher mobilization of Axin as a part of the degradation complex augmenting β-catenin turnover [65]. Using a murine model for adrenocortical carcinoma (ACC), Borges et al. showed that β-catenin activation cooperates with the loss of p53 promoting tumorigenesis [66]. In a study performed on prostate cancer tissues, mutations in Wnt/β-catenin signaling were less frequent in *TP53* mutant [67]. In a panel of HCC tissues, *CTNNB1* mutations were found to mutually exclusive to *TP53* mutations indicating that either of the two mutations might be adequate to initiate the carcinogenic process [68]. The emerging data suggests tissue-specific cancer mutations interplay in the WNT and p53 pathway. Recently crosstalk among WNT/β-catenin signaling and p53 has been extensively reviewed by Xiao et al. [69]. The authors suggest that crosstalk at various levels might result in positive as well as negative feedback loops and reciprocal transactivation which can lead to discrete cancerous phenotypes [69]. In contrast to p53, the other family members i.e. p63 and p73, have also been implicated in positive regulation of WNT/β-catenin signaling [70,71]. Differential expression of the p53 family gene members in various cancers and their bi-directional modulation of WNT/β-catenin signaling suggests a delicate equilibrium that needs to be further investigated.

With the advent of cancer genome databases, comprehensive and focused analysis of the mutation status of various tumor-associated genes is now possible. Large scale mutational setting of β-catenin was revealed in a Memorial Sloan Kettering-Integrated Mutation Profiling of Actionable Cancer Targets (MSK-IMPACT) study employing clinical sequencing of 10,000 prospective cancer patients [72]. A high frequency of *CTNNB*1 mutations was observed in endometrial (16%), hepatobiliary (12%), melanoma (7%), and colorectal (6%) cancers.

### 7.1. Colorectal cancers

The role of β-catenin in carcinogenesis was initially described and intensively studied in colon cancers. APC mutations are observed in over 80% of all Colon Cancers, which invariably exhibit cytosolic and nuclear accumulation of β-catenin.

In CRC, most somatic mutation hotspots are observed at Asp32, Ser33, Gly34, Ser37, Thr41, and Ser45 in exon 3 of β-catenin mRNA which stabilizes β-catenin by disturbing the phosphorylation-dependent degradation leading to carcinogenesis. Ser 45 is a priming phosphorylation site for CK1α; Ser33,Ser37, and Thr41 are phosphorylated by GSK3β, while D32 and G34 are required to bind with β-TrCP [15]. Yaeger et al. while studying metastatic CRCs reported 8% *CTNNB1* alterations with a high number of in-frame deletions in a targeted capture assay [73]. Activating hotspot mutations of β-catenin occurred more frequently in microsatellite instability-high or hypermutated tumors (25%) as compared to microsatellite stable tumors (6%). Large-in-frame deletions spanned exon 3 resulting in the elimination of regulatory phosphorylation sites in β-catenin preventing its degradation were identified in MSS tumors along with nuclear staining of β-catenin [73]. In a seminal study by Wong et al., the authors investigated the diagnostic and prognostic significance of nuclear β-catenin in colorectal cancer. They showed that compared to normal tissue (0%), polyps (8%), adenomas (92%), and carcinomas (100%) show significant nuclear β-catenin positivity. Moreover, high β-catenin scores were positively associated with lymph node metastasis and poor survival [74]. On the same line, recently Bhattacharya et al. showed higher β-catenin scores in poorly differentiated adenocarcinomas, when compared with well- or moderately- differentiated adenocarcinomas. Additionally, a higher nuclear β-catenin score was observed in mucinous adenocarcinomas, compared to adenocarcinomas NOS, suggesting its role in the worst prognosis in mucinous sub-type [75].

### 7.2. Endometrial cancer

β-catenin mutations could be potential prognostic markers in endometrial carcinoma. Integrated analysis revealed that *CTNNB1* exon 3 mutations are associated with an aggressive subtype of low-grade and low-stage endometrial carcinoma in younger women [76]. It was observed that exon 3 deletion in the *CTNNB*1 gene resulted in dysregulation of WNT/β-catenin signaling, and endometrial epithelial differentiation leading to hyperplasia in the murine model [77]. In the TCGA cohort of 240 endometrial cancer cases, alterations in *CTNNB1* or *APC* genes were observed to be 30% or 12% [78]. The specific mutation appears to occur at codon 34, causing changes at residues near Ser 33 leading to nuclear accumulation due to compromised degradation. Additionally, aberrant WNT signaling can also be evaluated using immunohistochemistry for β-catenin. Scholten et al. demonstrated that nuclear β-catenin immunopositivity is a molecular feature of type I endometrial carcinoma [79]. A separate study reported that high expression levels of β-catenin and the known WNT target genes *MYC* and *CCND1* were associated with worse overall survival in low-grade tumors [76]. Thus, in endometrial carcinoma, it seems that *CTNNB1* mutations, rather than APC mutations act as direct drivers rather than passengers. A study using 245 endometrial cancer patient samples by Kurnit et al. demonstrated that mutational analysis of *CTNNB1* gene could be helpful in the identification of low-grade, early-stage endometrial cancer patients with a higher risk of recurrence [80]. Among the 50 patients analyzed for *CTNNB1* mutation, 42 (84%) of the patients demonstrated β-catenin nuclear staining [80]. Furthermore, Kim et al. showed that β-catenin nuclear immunostaining exhibited 100% specificity in distinguishing *CTNNB1* mutant from wild type, though the sensitivity was on the lower side (84.9%). The authors recommended that immunohistochemistry could be the initial screen, and samples that are negative for β-catenin nuclear staining can be forwarded for *CTNNB1* sequencing [81]. On the contrary, a meta-analysis study by Travaglino et al. established nuclear β-catenin expression as a surrogate for Exon 3 *CTNNB1* mutation in endometrial cancers [82].

### 7.3. Breast cancer

β-catenin accumulation in the nuclei and/or cytosol of tumor cells has been observed in approximately 50–60% of human breast cancers and is associated with poor prognosis [83,84]. Mutations in *CTNNB1* gene, are relatively infrequent in breast cancer [85]. Activation of the WNT pathway is observed, however, via epigenetic activation of WNT components as well as the inactivation of WNT inhibitors [86,87]. Both canonical and non-canonical components of the WNT pathway are reported to be aberrantly expressed in breast carcinomas, primarily in the triple-negative and basal-like subtypes [88–90]. The genes that code for the destruction complex are frequently mutated, deleted, or hypermethylated which may or may not be aided by silencing or loss of E-cadherin in breast cancer, paving way for β-catenin nuclear translocation [91]. Both genetic and epigenetic alterations in APC have also been identified in human breast cancer. Specifically, in cases in which nuclear or cytosolic accumulation of β-catenin is observed at a higher frequency, or in the absence of specific β-catenin mutations, APC mutations or decreased expression have been identified. In colorectal cancer, where APC loss of heterozygosity is a very common and early event, mutations occur primarily in the mutation cluster region (MCR) in the middle of the coding region. In contrast, most APC mutations that occur in sporadic human breast cancer have been identified outside of the MCR [92,93].

The subtype-specific deregulation of WNT/β-catenin signaling may explain why some studies have failed to observe pathway activation or an association with any clinical parameters [94]. In any case, metaplastic and basal-like invasive breast carcinomas are aggressive, but poorly characterized on the molecular level and have minimal treatment options because of their triple-negative status. Therefore, the WNT/β-catenin pathway may offer a novel therapeutic target in these subclasses of breast cancers [93]. However, it is not yet fully known if this is related to the subtype-specificity observed with β-catenin cytosolic and nuclear accumulation [95], but this relationship is important to explore given that APC appears to have tumor suppressive activity in the mammary gland, and other tissues that are separate from regulating the WNT/β-catenin pathway [93,96,97].

**Figure 3.**
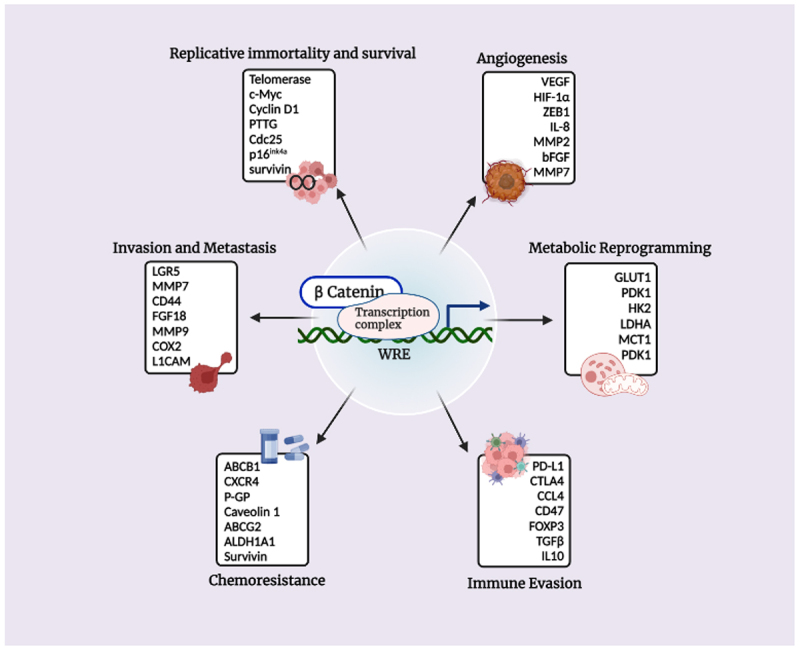
Transcriptional targets of β-catenin and their implications in promoting the hallmarks of cancer.

Notes: The figure encompasses a short list of genes that are under direct control of the β-catenin transcriptional activity which are broadly segregated based on their roles in promoting cancer progression and metastasis. It is noteworthy that the genes which are under transcriptional control of β-catenin have key roles to play in driving multiple hallmarks (i.e. replicative immortality and survival; angiogenesis; invasion and metastasis; metabolic reprogramming; chemoresistance; and immune invasion) of cancer making β-catenin a valuable therapeutic target. Created with BioRender.com

WNT/β-catenin signaling is involved in the pathogenesis of various cancers [98,99]. This pathway provides an opportunity to develop novel therapeutics for cancers by targeting the individual components or at distinct. Unfortunately, the development of therapeutics that specifically target WNT/β-catenin signaling in cancer has lagged due to its molecular characterization, and the development of specific animal models relevant to human disease [93]. A schematic diagram showing β-catenin transcriptional targets maneuvering different cancer hallmarks has been shown in Figure 3. This is in part because of the complicated nature of pathway regulation and crosstalk with other pathways, but also due to the well-conserved physiological roles of WNT/β-catenin signaling in normal cells. Detailed strategies for developing and screening WNT/β-catenin pathway inhibitors using biological assays have been reviewed extensively [93,100,101], while those with demonstrated anti-tumor activity will be discussed here.

## 8. β-catenin as a therapeutic target

Various components of the β-catenin signaling pathway are under intense investigation as potential therapeutic targets owing to their significant roles in multiple malignant programs including cancer stemness and metastasis. Various strategies have been developed to date for targeting β catenin including small molecules, antibodies, RNAi, and peptides [29,102]. Furthermore, an array of natural compounds has also been identified or isolated as inhibitors of β-catenin. Considering the readout of the oncogenic function of aberrant β-catenin expression is its transcriptional output, the pathway of β-catenin itself has been targeted at multiple points for therapeutic intervention.

Starting at the extracellular level, there is an opportunity to dampen the downstream signaling by targeting the WNT ligands themselves or by antibodies that selectively bind to the extracellular domains of receptor tyrosine kinases [103]. Restoration of sFRPs is also an arena that has been studied for its anti-tumor potential [100]. Moving further along the pathway, modulations of the regulatory proteins, more specifically the proteins of the β-catenin destruction complex also determine the signaling activity of β-catenin. Consistent with this, several molecules aimed at targeting GSK3, Tankyrases, Dsh, AXIN, Porcupine, CK1, etc. are being screened and tested for their clinical efficacy, case in point [101,104]. Having said this, a significant number of the clinical trials targeting WNT signaling are still in the early phases with unsatisfactory outcomes, often chalked up to compromised efficacy, specificity, and/or toxicity. The strategies aimed at upstream components of the pathway, especially at the receptor, enzyme, or transduction-level lack specificity owing to the cross-regulatory effects on other functions and pathways. Also, there is a legitimate concern that downstream activating mutations could render them ineffective. Furthermore, the ‘ON’ and ‘OFF’ target side effects and toxicity on the normal cells especially structural integrity is also a cause of concern [105]. For instance, many recorded targeted therapies suggest that acute inhibition resulted in significant deterioration in bone density, considering the pivotal roles played by the WNT pathway in bone homeostasis [106]. Hence, there have been efforts in targeting the downstream components, or more specifically, β-catenin in the nucleus as this would render concerns regarding the mechanisms of activation redundant.

### 8.1. Manoeuvring down the slippery slope

To comprehend the prospects of directly targeting β-catenin, we must first re-acquaint ourselves with the dynamics of its structure. As discussed earlier, most of the protein–protein interactions (PPIs) of β-catenin happen via the *armadillo* domain where the major-binding partners include TCF3, TCF4, LEF1, BCL9, AXIN, APC, E Cadherin, etc. Structurally, two key challenges complicate the possibility of therapeutically targeting β-catenin:

1. *Overlapping PPI interfaces*: A successful targeting of the β-catenin protein would entail the disruption of its interaction with its transcriptional partners while leaving the E cadherin/APC interaction unperturbed. This is because the PPI with cadherin is necessary for proper maintenance of cell–cell adhesion and epithelial phenotype while the APC interaction is crucial in moderating β-catenin degradation [107]. Disrupting either of these may have far-reaching consequences since any drug administered might adversely affect the normal epithelial cells in addition to targeting transformed cells.
2. *The protein–protein interaction between β-catenin and the TCF/LEF members*: TCF3 and TCF4 primarily bind along the positive charge groove of the *armadillo* superhelix [108]. The area of this interface is about 4500 A^2^ which is atypically large and hence a challenging hurdle. The K_D_ of this interaction is estimated to be around 10 nM, which is significantly lower compared to catenin-cadherin and catenin–APC interactions that fall in the range of ≤82 nM and ≤ 3.1uM respectively [107]. Adding to this is the ‘multisite’ nature of the β-catenin-TCF4 hydrophobic interactions. The area of interaction paired with the unusually low K_D_ makes it a nonviable target in terms of achieving inhibitor specificity and as a consensus considered a dead end in terms of inhibitor development, since any inhibitor should be able to overcome the high affinity of this bond.

### 8.2. Inhibitors of the β-catenin PPIs

The landscape of this so-called ‘un-targetability’ of β-catenin was changed by Shivadasani’s lab in 2004, wherein a seminal screening with about 7000 compounds was focused on achieving reproducible and dose-dependent inhibition of the β-catenin-TCF4 PPI. The screening revealed eight different compounds – PKF115–584, CGP04090, PKF222–815, PKF118–744, PKF118–310, ZTM000990, NPDDG39.024, and NPDDG1.024. Of these two compounds, CGP04090 and PKF115–584 showed limited toxicity, while inhibiting β-catenin in Xenopus embryo *in vivo* [109]. A major drawback of these compounds is that the structural mechanism of inhibition is still largely unclear. Also, these compounds contained functional groups like quinone or taxoflavin which are widely recognized as substructures of the pan assay interference compounds (PAINS) that are reported to cause false positives in biochemical assays [107].

A noteworthy observation concerning the β-catenin -TCF PPI is that despite the large area of interface of the two proteins, there exists a few key residues at the interface or ‘hotspots’ that contribute to the major share of the binding free energy and a mutation or disruption of the interaction of these particular residues will interfere significantly with the binding affinity of the proteins [110]. Over the last two decades, about four different regions of the β-catenin structure were revealed to be instrumental in modulating its interaction with TCF. Region 1 contains K435, K508, N426, R469, and H470, region 2 contains K312 and K345, the hydrophobic region 3 contains residues F253, I256, F293, A295, and I296; and region 4 contains residues H578 and R582 [107,111].

The first study that targeted the hotspot regions of β-catenin was done by Trosset et al., as they employed *in silico* docking paired with biophysical screening to look for molecules that targeted the K435 and R469 hotspot region of β-catenin -TCF PPI. About 17,700 compounds were screened of which PNU-74654 was identified as a prospective candidate [112]. Further investigations on the selectivity and molecular activity were not reported for this molecule which might in part be because of the presence of an acyl hydrazone moiety (another PAINS substructure). In another study, RNAi-based knockdown of AXIN in combination with integrated screening technology identified three compounds – iCRT3, iCRT5, and iCRT14 which were inhibitors of catenin-responsive transcription (CRT) [113]. In a study by Grossman et al., hydrocarbon stapled peptides StAx-35 and StAx-35 R were designed to model the AXIN sequence which was successfully found to co-crystallize with β-catenin [114]. The structure of the peptide was further optimized by adding the NLS of the SV40 large T antigen and a replacing all arginine residues with homoarginines to construct NLS-StAx-h [115].

**Figure 4.**
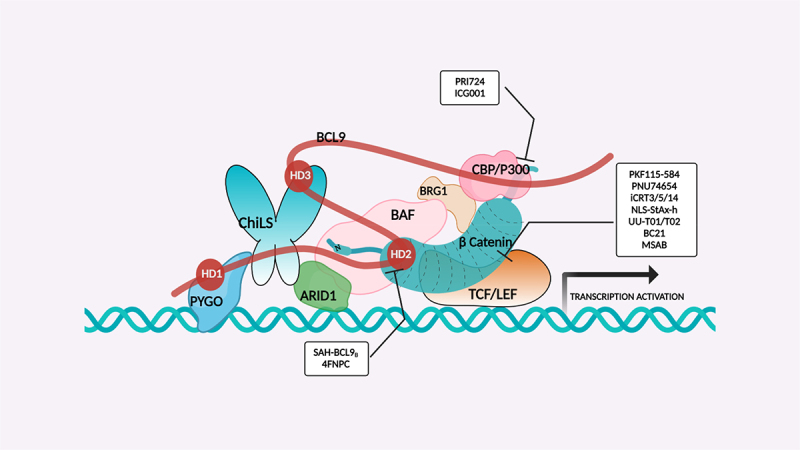
β-catenin enhanceosome complex and therapeutic alternatives.

Notes: A schematic illustration of the β-catenin transcriptional complex or the ‘enhanceosome.’ The inhibitors that target β-catenin and perturbs its interaction with BCL9, TCF, and CBP are included in respective boxes. A detailed list of β-catenin inhibitors and their mode of action is provided in Tables 1 & 2. Created with BioRender.com

TCF-dependent luciferase reporter system was used to scan a library of 22,000 compounds by Hwang et al., which resulted in the identification of MSAB [116]. Biotin-based affinity purification verified its binding to β-catenin and it has been shown to successfully downregulate WNT target genes. To target the β-catenin-BCL9 interaction, a peptide sequence SAH-BCL9_B_ was developed which displayed competitive inhibition by mimicking the alpha helical domain of BCL9 protein [117]. A comprehensive list of β-catenin inhibitors can be found in Table 1. Schematic diagram demonstrating β-catenin inhibitors and their binding sites in β-catenin enhanceosome has been shown to Figure 4.

**Table 1.** A comprehensive list of β-catenin inhibitors with structures and mode of action.

S. No.	Inhibitor Name	Chemical Structure	Mode of Action	Reference
1	PKF115–584	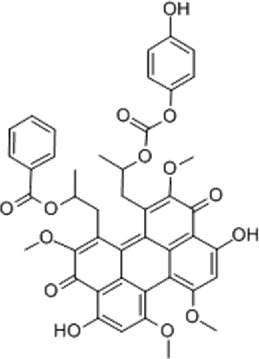	Disrupts β-catenin/TCF interaction	[109,118]
2	PNU-74654	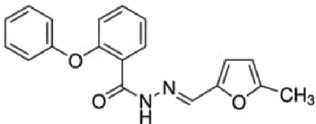	Binds to K435/R469 hotspot of β-catenin, and disrupts TCF interaction	[112]
3	PKF118–744	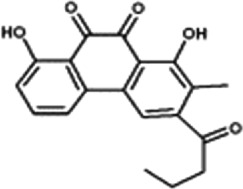	Inhibits β-catenin/TCF interaction	[109]
4	CGP049090	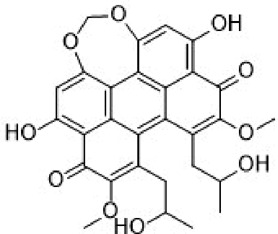	Interferes with β-catenin/TCF interaction	[109,112]
5	PKF118–310	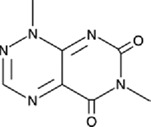	Obstructs in β-catenin/TCF interaction	[109,112]
6	ZTM000990	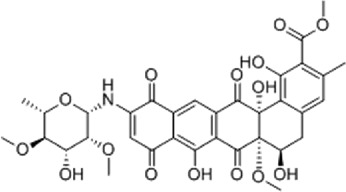	Disrupts the β-catenin/TCF interaction	[109,112]
7	BC21	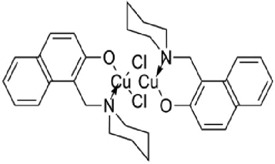	Interferes with TCF-4/β-catenin binding	[119]
8	CCT036477	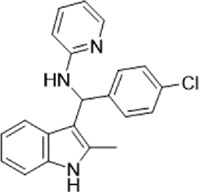	Inhibits TCF dependent transcription by preventing β-catenin stabilization	[120]
9	PKF222–815	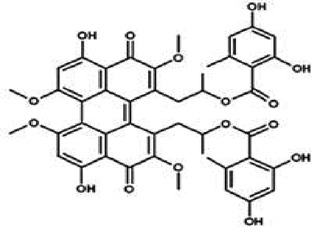	Disrupts the β-catenin/TCF binding	[109]
10	CWP232228	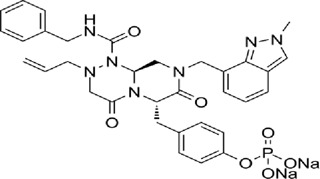	Antagonizes β-catenin binding to TCF-4	[121]
11	PRI-724/C-82	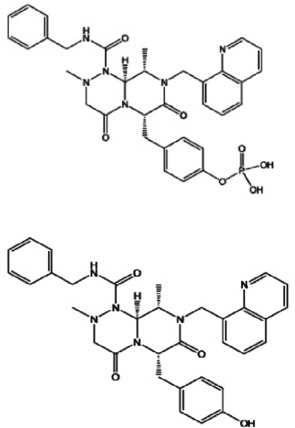	Blocks CBP-β-catenin interaction	[122]
		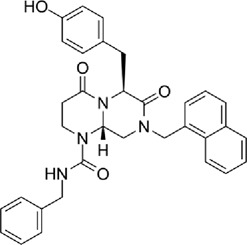		
12	ICG001	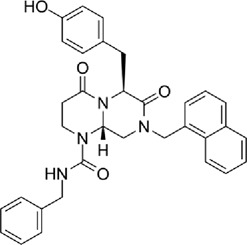	Blocks CBP-β-catenin interaction	[122,123]
13	MSAB	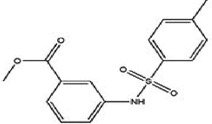	Binds to β-catenin and promotes its proteasomal degradation	[116]
15	SAH-BLC9_B_	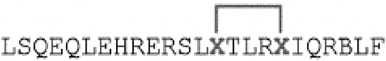	Disrupts BCL-9/β-catenin complex	[117,124]
16	ZINC02092166	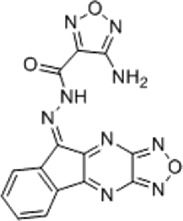	Inhibits β-catenin/TCF PPI	[107]
17	iCRT3	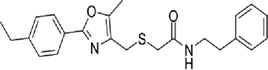	Inhibits β-catenin/TCF interaction	[113,119]
18	iCRT5	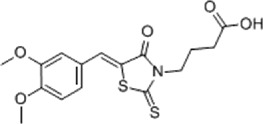	Inhibits β-catenin/TCF interaction	[113]
19	iCRT14	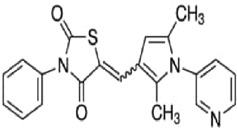	Inhibits β-catenin/TCF interaction	[113]
20	NLS-StAx-h	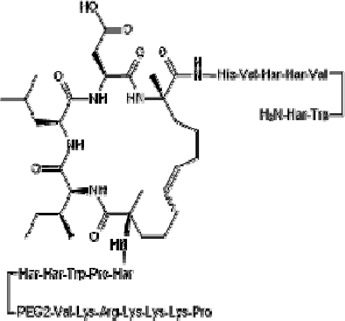	Disrupts β-catenin PPI with TCF/LEF	[115]
21	H1-B1	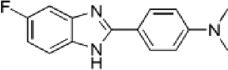	Inhibits β-catenin/TCF-4 interaction	[125]
22	UU-T01, T02	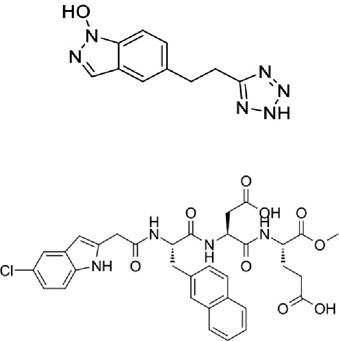	Inhibits β-catenin/TCF PPI	[126]
23	4FNPC	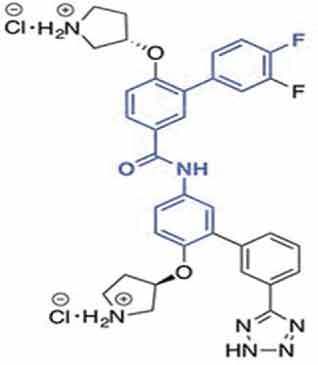	Disrupts β-catenin/BCL-9 complex	[127]

**Table 2.** A comprehensive list of β-catenin natural inhibitors along with the structure and mode of action.

S. No.	Inhibitor Name	Chemical Structure	Mode of Action	Reference
1	Apigenin	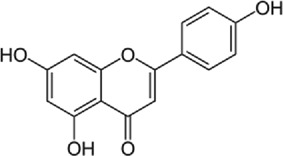	Obstructs the β-catenin/TCF-mediated transcriptional activity	[148]
2	Carsonic acid	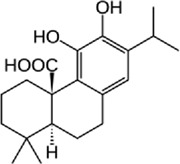	Interferes with BCL9-binding site in β-catenin	[147]
3	Curcumin	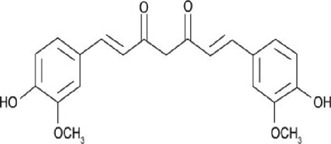	Inhibits β-catenin nuclear translocation of β-catenin/TCF binding to the promoter DNA.	[137,138,139]
4	Esculetin	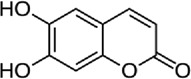	Disrupt the β-catenin-Tcf complex	[146]
5	Magnalol	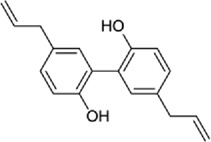	Reduces β-catenin nuclear translocation and interferes in β-catenin/TCF complexes to their respective DNA-binding sites	[149]
6	Resveratrol	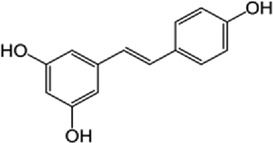	Inhibits the β-catenin/TCF4 interactions and facilitate the TCF4 proteasomal degradation	[140,141]
7	Silibinin	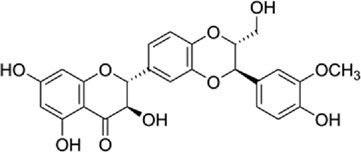	Interferes β-catenin and TCF interaction	[144]
8	Toxoflavin	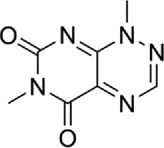	Antagonist of transcription factor 4 (TCF4)/β-catenin complex	[139]

## 9. miRnas mediated therapeutics for β-catenin

As discussed, WNT/β-catenin plays a crucial role in the cancer progression and metastasis. Previous studies have established the positive role of WNT/β-catenin signaling in giving rise to EMT process, which is characterized by the nuclear translocation of β-catenin protein in transformed cells thereby suppressing the E-cadherin protein. This loss of E-cadherin results in the dissociation of cell–cell adhesion through which cancer cells gain motility. Various EMT-inducing transcription factors, which are downstream to β-catenin have been shown to suppress E-cadherin directly are Twist, Snail, Slug, and ZEB and are also believed to be controlled by MicroRNAs (miRNAs) in various cancers [118]. miRNAs are small non-coding sequences (19–25 nucleotide) that modulate the gene-expression post-transcriptionally thereby regulating cellular and physiological processes, i.e. cell differentiation and homeostasis [119,120]. Various studies documented that miRNA may function as inhibitors of EMT process by targeting the WNT pathway or its downstream targets. In consequence, overexpression of these miRNAs may work as therapeutic strategy to circumvent EMT process. Among diverse microRNAs, miR-200 family has been shown to impair WNT signaling by binding to β-catenin transcripts and suppress its translation [121]. Additionally, it also represses the transcription factor ZEB1/2. miR-200 mediated effect on ZEB and β-catenin has been documented in various cancers [122–125]. Additionally, overexpression of miR-200 has been shown to induce E-cadherin upregulation thereby gaining epithelial characteristics in cancer cells, due to direct targeting of ZEB1/2 [126,127]. Another miRNA, miR-33b also binds to the 3’-UTR region of ZEB1 thereby impairing the β-catenin-induced EMT in lung adenocarcinomas [128]. Other miRNAs like miR33a, miR-34b/c and miR-3619-5p have been shown to target the 3’UTR region of β-catenin thereby blocking EMT in various cancer [129–131]. miR-203 suppresses Slug, a transcriptional suppressor of E-cadherin via GSK3/β-catenin axis in prostate cancer [132]. Recently, miR-5188 was shown to modulate the nuclear β-catenin expression via FOXO1 thereby inducing EMT and cancer stemness in HCC and breast cancer [133]. The detailed interaction between WNT/β-catenin pathway and miRNA network has been recently reviewed by Lei et al. [134]. Overall, these studies clearly suggest that β-catenin mediated transcription downstream of WNT signaling can be therapeutically modulated either by restoring miRNA expression or by inhibiting miRNA function that might lead to the development of efficient cancer treatment strategies.

## 10. Natural compounds targeting β-catenin

β-catenin protein is now well-established as a cancer promoter, and it has been demonstrated as a potential therapeutic target for anti-cancer strategy [114,135]. In this context, multiple natural compounds have been shown to modulate or impair β-catenin activity either by downregulating the β-catenin itself, or its coactivators, hence affecting the downstream target genes [136]. Curcumin, the renowned chemopreventive agent extracted from the rhizome of the *Curcuma longa* plant has been shown to possess anti-inflammatory, neuroprotective, and antioxidant activity. In cancers, curcumin has been shown to affect various oncogenic signaling, in particular WNT/β-catenin signaling. In breast cancer cells, we have previously shown that curcumin effectively impairs the expression of several WNT/β-catenin signaling components viz. E-cadherin, Dishevelled, GSK3β, β-catenin, Cyclin D1, and Slug [137]. Curcumin has also been shown to inhibit the β-catenin nuclear translocation by disturbing the β-catenin/TCF binding to the promoter DNA, thereby inhibiting the downstream targets of β-catenin affecting cell proliferation, migration, and invasion of cancer cells [138,139]. Authors demonstrated that magnolol inhibits the proliferation and invasion potential of colorectal cancer cells both *in vitro* and *in vivo* by suppressing the β-catenin nuclear translocation, and interfering with β-catenin/TCF complexes to their respective DNA-binding sites. Resveratrol, a flavonoid also inhibits the β-catenin/TCF4 interactions and facilitates the TCF4 proteasomal degradation [140,141]. Furthermore, low-dose resveratrol inhibited the colon cancer cells by suppressing β-catenin through legless (*lgs*) and Pygopus I and II (*pygoI*, *pygoII*) [142]. Another natural compound, Lonchocarpin (a chalcone) isolated from *Lonchocarpus Sericeus* inhibited activation of β-catenin induced through either the wild-type β-catenin, β-catenin(S33A) of dn TCF4 VP16 by impairing the TCF4 mediated transcription [143]. The authors of the study advocated that lonchocarpin can be a possible clinical trial candidate over PRI-724, as the clinical trial for the latter has been withdrawn because of supply issues (NCT02413853). Silibinin, an active ingredient of milk thistle (*Silybum marianum*) extract demonstrated the downregulation in β-catenin transcriptional activity in human colorectal SW480 cells [144]. Silibinin not only decreased the TOP FLASH reporter activity but also resulted in a reduction of CDK8 and Cyclin C thereby demonstrating its mode of action through interfering β-catenin and TCF interaction. Authors further showed a decrease in β-catenin levels in parallel with inhibition of tumor growth generated via SW480 ×enografts in nude mice. Similar in renal cell carcinoma (RCC) cells i.e. 786-O, silibinin suppressed TOP FLASH reporter activity to 50%, thereby demonstrating silibinin’s potential to inhibit β -catenin transcription in RCCs thereby inhibiting epithelial-to mesenchymal transition through autophagy [145]. Esculetin (6,7-dihydroxycoumarin or esculetin) is a bioactive component of *Fraxinux rhynchophylla* that has been shown to disrupt the β-catenin-TCF complex (by direct binding with the Lys312, Gly307, Lys345, and Asn387 residues) of the β-catenin protein in colon cancer cells. Esculetin effectively antagonized the β-catenin-dependent activity and suppressed the growth in a colon cancer xenograft tumor in mouse model [146]. In a screening for small compounds that inhibited the β-catenin’s binding to its cofactor BCL9, La Roche *et al*, found that carsonic acid (from rosemary) inhibits the transcriptional β-catenin in colon cancer cells. Using NMR and analytical ultracentrifugation, the author demonstrated that carnosic acid riposte depends on an intrinsically labile α-helix (H1) amino-terminally juxtaposed the BCL9-binding site in β-catenin [147]. The other potential natural transcriptional inhibitors of β-catenin protein than can be further studied for their role in anti-cancer therapy are: Apigenin [148]; Toxoflavin (PFK118–310) [139]; Magnolol [149]; and 2-Hydroxycinnamaldehyde [150]. A comprehensive list of β-catenin natural inhibitors can be found in Table 2.

## 11. Targeting nuclear β-catenin

The development of cancer is characterized by an abnormal and uncontrolled proliferation of cells. EMT, stemness, angiogenesis, modification in the tumor microenvironment, and immunomodulation are essential tactics employed by the cells to fuel their aggressiveness, which eventually leads to metastasis and therapy resistance. WNT/β-catenin pathway has been implicated in all these phenomena and hence put forward its significance in the context of cancer therapeutics. However, the idea that activation of the WNT pathway is restricted to a linear or sequential series of events that happens upon receptor activation by WNT ligands is an oversimplification. The pathway features a network of interconnected interactions and the present understanding of the mechano-transduction is yet to reach the temporal and molecular resolution to comprehend the exact dynamics of its complex pathophysiology. Also, though β-catenin is often (wrongly) used synonymously with the WNT pathway, as it is regulated by an array of other molecules, pathways, and network crosstalk’s that cancer cells oftentimes take advantage of this malleability to propel its growth and metastasis. From a therapeutic point of view, this interplay provides multiple potential targets for intervention.

Many compelling strategies have been proposed and executed starting at the level of WNT ligands, with the goal of inhibiting nuclear β-catenin and consequently its transcriptional activity. The landscape of WNT therapeutics, however, has witnessed a gradual shift in focus from upstream targeting to the targeting of downstream components or more specifically, nuclear β-catenin itself and its interaction with transcriptional partners. Traditionally, the protein has been considered undruggable due to the peculiarity of its structure but this myth of ‘undruggability’ has been challenged successfully by the design of small molecules, peptide sequences, and even natural compounds that have been demonstrated to target β-catenin PPI with its partners as discussed in the present review. Many of these molecules are identified through virtual docking experiments and other *in silico* screening approaches. Rational drug design has also been employed, which has led to the development of molecules like UU-T01, UU-T02, HI-B1, 4FNPC, etc., and more interestingly, β-catenin targeting peptides like NLS-Stax-h and SAH-BCL9 [115,117,151–154]. The clinical experience of β-catenin targeting drugs however is still incipient.

## 12. Future perspectives

Our evolving understanding regarding the intricacies associated withβ-catenin structure and functions has opened several new windows of opportunity. A few plausible but relatively untraversed strategies are discussed briefly below:

- *The terminal domains of β-catenin*: Most of the therapy design so far for β-catenin has been concentrated on a few hotspot regions and residues present in the central *armadillo* domain, while leaving the amino and carboxy domains largely unexplored due to their intrinsically disordered nature. While the terminal domains have a major functional contribution; very little is known in the way of their crystal structure because of their inherent conformational heterogeneity and highly dynamic secondary structures. Emerging strategies of *in silico* simulation studies and atomic characterization employing spectroscopy have the scope to provide information on their dynamic conformational ensembles. Also, the potential of stabilizing the secondary structures of these domains via small molecules/peptides cannot be disregarded either.
- *‘Cloud’ inhibition and combination therapy*: Of note, WNT pathway deregulation is accompanied by simultaneous mutations/deregulations in other pathways. Hence, targeting isolated molecules might not be significant from a therapeutic point of view as the ‘extent’ of pathway impediment or suppression of tumor progression achievable by downregulating a single molecule is debatable. A more pragmatic approach would be to target the imbalanced pathway at different points concomitantly [155]. This ‘Cloud’ inhibition is more relevant in the case of β-catenin, since multiple pathways and molecules regulate/or are regulated by the protein itself. Additionally, it is warranted that future studies should try and test β-catenin targeted therapy in combination with other targeted agents/or standard chemotherapy, as opposed to reliance on monotherapy.
- *Phytochemicals and repurposed drugs*: As discussed extensively in the review, manyβ-catenin targeting molecules have been identified (including natural products). Natural products viz. flavonoids, sterols, lignins, and other polyphenolic compounds are shown to regulate β-catenin function by modulating the intracellular β-catenin levels through inhibition of its expression, expediting its degradation, regulating its phosphorylation status, and controlling its nuclear import. These phytochemicals offer an attractive advantage of serving as templates that can be used to streamline drug design that offers enhanced specificity and reduced toxicity. Apart from phytochemicals, drug repurposing has also gained attention considering drug discovery being time-consuming and monetarily taxing. A selected few NSAIDs (Non-Steroidal Anti-Inflammatory Drugs), antimicrobials, antiparasitics, etc., have shown potential to modulate the WNT signaling components and they hold much prospect as well.
- *Fundamental mechanistic studies*: The solution to safely targeting β-catenin lies in unraveling the pleiotropy of the molecule. For successful translation to the clinic, fundamental studies are warranted in the following areas i.e. spatio-temporal resolution of the nuclear import of stabilized pleiotropy; tissue-specific interactors of the β-catenin protein and its influence on the transcriptional output; characterizing the interacting partners and target proteins of β-catenin that influences its role in enhancing stemness competence and plasticity of cancer cells.

It is also worth mentioning that most of the drug target interactions of β-catenin inhibitors have so far been studied via closed/or *in vitro* systems. The lifetime and efficacy along with the thermodynamics and kinetics of drug–target interactions in open/or *in vivo* systems vary drastically where the drug concentration and permeability fluctuate over time, affecting its *in vivo* potency. Acknowledging β-catenin’s role in normal cells, a safe targeting also entails minimal toxicity. To this end, future studies should also prioritize optimizing pharmacodynamic factors of the inhibitors like half-life, retention time, and renal threshold, which can have dramatic effects on the safety and efficacy of drugs.

## 13. Conclusions

Tissue specificity of WNT pathway alterations and the possibility of a tissue-dependent predisposition in the transcriptional output of β-catenin are questions that still lack definitive answers. Thus, the versatility of β catenin has proven to be a double-edged sword due to which β-catenin targeted therapeutics have to undergo a robust clinical translation and validations. It would be interesting to explore the context-dependent differences in pharmacological and signaling dynamics brought upon by β-catenin inhibition. With enough deliberation, therapeutic targeting of β-catenin might address concerns of stemness, plasticity, and resistance of malignant cells, which remain elusive to conventional chemotherapy.

## Data Availability

Data sharing is not applicable to this article as no new data were created or analyzed in this study.

## Abbreviations

Wg, Wingless; APC, Adenomatous Polyposis Coli; TCF, T-cell factor; LEF, lymphoid enhancer factor; GSK3β, Glycogen Synthase Kinase 3β; CK1α, Casein Kinase 1α; AXIN, APC axis inhibitor; β-TrCP, β-transducin repeat containing protein; Skp1, S-Phase Kinase Associated Protein 1; EDD, E3 Ubiquitin Ligase identified by Differential Display; GID complex, Glucose-Induced Degradation (GID) ubiquitin ligase complex; Jade1, Gene for Apoptosis and Differentiation (Jade) Family PHD Finger 1; Mule, Mcl-1 Ubiquitin Ligase E3; SHPRH, SNF2 Histone Linker PHD RING Helicase; SIAH1, Seven In Absentia Homolog (SIAH) E3 Ubiquitin Ligase 1; LRP5, Low-Density Lipoprotein receptor-related protein 5; FOXM1, Forkhead Box Protein M1; PAK4, P21 (RAC1) Activated Kinase 4; BCL9, B-Cell CLL/Lymphoma 9; CBP,CREB-binding protein; BRG1, Brahma-Related Gene 1; CDKN1A, Cyclin Dependent Kinase Inhibitor 1A; FOXO, Forkhead Box O; HIF1α, Hypoxia-Inducible Factor 1 alpha; Sox, SRY-Related HMG-Box Genes; IGF, Insulin-like Growth Factor; FGF, Fibroblast Growth Factor; MEKK2, Mitogen-Activated Protein Kinase Kinase 2; USP15, Ubiquitin Specific Peptidase 15; PTEN, Phosphatase and Tensin Homolog.

## Highlights

- β-catenin is crucial in oncogenesis through its dual role in adhesion and signaling.
- Its aberrant expression is an established prognostic and diagnostic marker.
- β-catenin targeted therapy is yet to be effectively translated from bench to bedside.
- Advances in mechanistic studies might open new windows of opportunity.
